# Profiling of snoRNAs in Exosomes Secreted from Cells Infected with Influenza A Virus

**DOI:** 10.3390/ijms26010012

**Published:** 2024-12-24

**Authors:** Wojciech Rozek, Malgorzata Kwasnik, Wojciech Socha, Bartosz Czech, Jerzy Rola

**Affiliations:** 1 Department of Virology, National Veterinary Research Institute, 24-100 Pulawy, Poland; malgorzata.kwasnik@piwet.pulawy.pl (M.K.); wojciech.socha@piwet.pulawy.pl (W.S.); jrola@piwet.pulawy.pl (J.R.); 2 GeneStat Bartosz Czech, 51-503 Wroclaw, Poland; bartosz.czech@icloud.com

**Keywords:** influenza virus, exosomes, non-coding RNA, snoRNA, TF, RBP

## Abstract

Small nucleolar RNAs (snoRNAs) are non-coding RNAs (ncRNAs) that regulate many cellular processes. Changes in the profiles of cellular ncRNAs and those secreted in exosomes are observed during viral infection. In our study, we analysed differences in expression profiles of snoRNAs isolated from exosomes of influenza (IAV)-infected and non-infected MDCK cells using high-throughput sequencing. The analysis revealed 133 significantly differentially regulated snoRNAs (131 upregulated and 2 downregulated), including 93 SNORD, 38 SNORA, and 2 SCARNA. The most upregulated was SNORD58 (log2FoldChange = 9.61), while the only downregulated snoRNAs were SNORD3 (log2FC = −2.98) and SNORA74 (log2FC = −2.67). Several snoRNAs previously described as involved in viral infections were upregulated, including SNORD27, SNORD28, SNORD29, SNORD58, and SNORD44. In total, 533 interactors of dysregulated snoRNAs were identified using the RNAinter database with an assigned confidence score ≥ 0.25. The main groups of predicted interactors were transcription factors (TFs, 169 interactors) and RNA-binding proteins (RBPs, 130 interactors). Among the most important were pioneer TFs such as POU5F1, SOX2, CEBPB, and MYC, while in the RBP category, notable interactors included Polr2a, TNRC6A, IGF2BP3, and FMRP. Our results suggest that snoRNAs are involved in pro-viral activity, although follow-up studies including experimental validation would be beneficial.

## 1. Introduction

The advancement of high-throughput sequencing technologies has unveiled the complexity and diversity of the eukaryotic transcriptome. Although most genomic DNA undergoes transcription, only an estimated 1–2% of the transcriptome encodes functional proteins, with the remainder comprising various non-coding RNAs (ncRNAs) [1]. These ncRNAs, through their interactions with other biomolecules, play pivotal roles in gene regulatory networks [2]. Based on functionality, ncRNAs can be categorised into regulatory and housekeeping types. Regulatory RNAs, including microRNAs (miRNAs), long non-coding RNAs (lncRNAs), circular RNAs (circRNAs), PIWI-interacting RNAs (piRNAs), and Y RNAs, influence gene expression and transcript stability [3]. Housekeeping ncRNAs are essential for fundamental cellular functions and maintenance, encompassing ribosomal RNA (rRNA), transfer RNA (tRNA), small nuclear RNA (snRNA), small nucleolar RNA (snoRNA), and telomerase RNA (TERC) [4]. Although snoRNAs are primarily classified as housekeeping ncRNAs due to their crucial role in modifying rRNA and tRNA, recent studies have shown that some snoRNAs also perform regulatory functions, impacting gene expression and other cellular processes beyond their traditional roles. Typically 60–300 nucleotides long and enriched in the nucleolus, snoRNAs are components of ribonucleoprotein complexes (snoRNPs), where they serve as scaffolds for proteins and guide snoRNPs to target RNAs via base-pairing mechanisms. SnoRNAs are divided into two major types: C/D box snoRNAs (SNORD), involved in 2’-O-methylation, and H/ACA box snoRNAs (SNORA), which mediate the pseudouridylation of uridines in target sequences. A subclass known as small Cajal body-specific RNAs (SCARNAs) exhibits features of both C/D and H/ACA box snoRNAs and participates in snRNA modification [5,6]. Sequence identity and conservation across species aid in identifying snoRNA copies and defining snoRNA families. Similar sequences within a family are typically designated with differing suffixes, such as SNORA2A, SNORA2B, and SNORA2C. Additionally, snoRNAs can be processed into smaller fragments, called sno-derived RNAs (sdRNAs) [7,8], which increases during non-optimal conditions, suggesting potential roles in stress regulation [9]. Beyond their canonical functions, snoRNAs are also involved in the regulation of mRNA abundance, alternative splicing, protein binding, polyadenylation, and chromatin decondensation. The dysregulation of snoRNAs has been linked to cancers, Alzheimer’s disease, and genetic disorders such as Prader–Willi syndrome [9,10]; they may also influence the course of viral infections [8,11,12,13].

An increasing number of studies are highlighting the role of snoRNAs in viral infections. Knockdown studies have confirmed the involvement of specific snoRNAs in viral replication, suggesting that RNA viruses use them to regulate gene expression. As described previously, snoRNAs may be important due to their effect on the mRNA “cap-snatching” phenomenon involving the cleavage of the 5’-terminal fragments of host pre-mRNA required to initiate the transcription of viral mRNA, which is associated with host protein synthesis “shut-off” [14,15]. These mechanisms are especially important during influenza virus infections, and the role of snoRNAs in these processes represents an interesting research trend.

SnoRNAs can be transported via extracellular vesicles (EVs), whose contents vary depending on the biological state of the source cell [16]. In general, the different RNAs including snoRNAs are selectively packaged into exosomes. Once translocated, they perform functions in target cells that not only reflect their intrinsic role in the original cell but also engage in novel mechanisms of action. These extracellular RNAs influence the behaviour of individual immune cells and contribute to both local and systemic immune responses. The impact of RNA-mediated communication on immune cells and disease states has significant implications for developing new disease biomarkers and creating innovative therapies for immune-related disorders [17]. The identification of dysregulated snoRNAs in exosomes during influenza infection may open up new applications, providing insights into virus–host interactions and the way for new strategies in diagnosis, treatment, and immune modulation. If specific exosomal snoRNAs are found to be essential for viral replication or survival, they could become promising targets for antiviral drug development. This could be achieved using small interfering RNAs (siRNAs) or antisense oligonucleotides, offering a new class of RNA-based therapeutic agents capable of reducing viral load [18]. Recent studies suggest that snoRNAs may also play a broader role in anti-cancer treatments, including targeted therapies and immunotherapies [19]. However, there is currently limited information on the potential of snoRNAs as therapeutic targets in viral infections, highlighting an important area for future research. Studying snoRNAs in exosomes offers unique opportunities to uncover novel mechanisms of intercellular communication, which complement and extend insights gained from studying them within cells. Exosomes can serve as natural delivery vehicles for snoRNAs for targeted therapies.

It was observed that West Nile Virus (WNV) infection significantly changed the levels of certain host miRNAs, small ncRNAs and mRNAs incorporated into EVs. Functional classification of RNAs differentially incorporated into EVs upon infection demonstrated an association of them with viral replication and proinflammatory pathways. Additionally, it was proven that both IFN-dependent and IFN-independent processes are involved in the regulation of RNA sorting into EVs during infection [20].

Influenza virus replicates entirely within the nuclei of host cells, increasing the likelihood of interactions with snoRNAs [21]. In previous studies, we isolated two exosome populations from Influenza A virus (IAV)-infected and uninfected Madin–Darby canine kidney (MDCK) cells using ultracentrifugation following adsorption with chicken erythrocytes as a purification step. Using next-generation sequencing (NGS), we identified differentially expressed protein-coding RNAs between these two groups of exosomes [22]. In the present study, we analysed the snoRNA profile of exosomes secreted during IAV infection. We performed the analysis of interactions of snoRNA differentially expressed in exosomes secreted by IAV-infected cells using the RNAinter algorithm tool [23]. This study aimed to investigate how IAV infection may alter the snoRNA composition of exosomes and explore the potential relevance of differentially expressed snoRNAs and their interactions in the mechanism of influenza virus replication.

## 2. Results

### 2.1. snoRNA Dysregulation Analysis

In this study, we characterised the snoRNA composition of exosomes derived from IAV-infected and mock-infected MDCK cells. In this group, we identified 131 upregulated and 2 downregulated snoRNAs. In the current study, we annotated snoRNAs into specific categories using the Rfam 14.10 database, which provides comprehensive information on RNA families [24]. Specifically, the majority of dysregulated snoRNAs were categorised into two main classes: SNORD and SNORA, corresponding to the two major types: C/D box and H/ACA box, respectively; additionally, two snoRNAs were categorised into the SCARNA group, which combines both activities (Table 1, Table S1).

**Figure 1:**
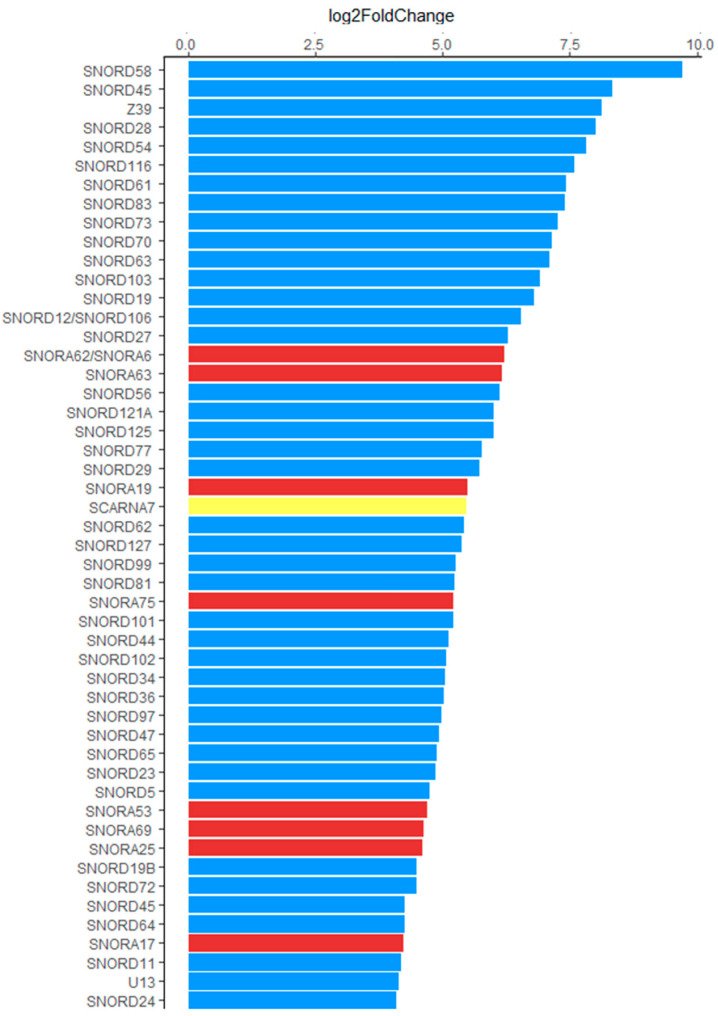
List of the 50 most upregulated snoRNAs, ranked by their log2Fold Change values. Colours indicate the classification of upregulated snoRNAs into specific types: C/D box snoRNAs (SNORD)—blue, H/ACA box snoRNAs (SNORA)—red, and SCARNAs—yellow.

SnoRNAs were subsequently ranked according to the log2FoldChange (log2FC) of their expression level. Among the top 50 most upregulated snoRNAs, the majority (41), represented the SNORD class, with log2FC values ranging from 4.05 (SNORD24) to 9.61 (SNORD58). In comparison, there were only eight SNORA in this group, with log2FC ranging from 4.58 (SNORA69) to 6.15 (SNORA62/SNORA6), and only one SCARNA (SCARNA7, log2FC = 5.40) (Figure 1). The only snoRNA identified as downregulated in exosomes from IAV-infected cells were SNORA74 (log2FC = −2.67) and SNORD3 (log2FC = −2.98).

### 2.2. snoRNAs Interaction Analysis

**Figure 2:**
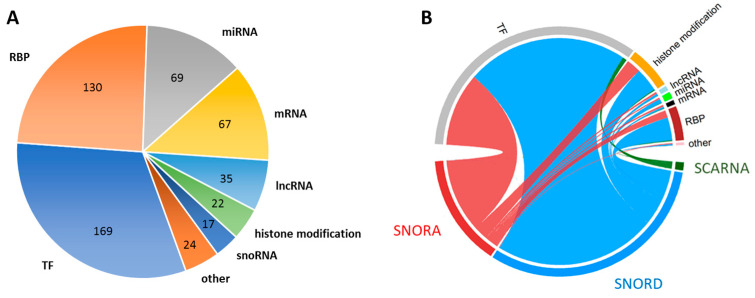
Graphical representation of snoRNA interactions: (**A**). Main categories of predicted interactors for dysregulated snoRNAs. Groups of interactors within each category are marked with colours: transcription factors (TF)—blue, RNA-binding proteins (RBP)—orange, microRNAs (miRNA)—grey, messenger RNAs (mRNA)—yellow, long non-coding RNAs (lncRNA)—light blue, histone modifications—green, snoRNAs—dark blue, and others—dark orange. (**B**). A circus plot illustrating the proportion of predicted interactions between various types of snoRNAs and the main categories of interactors. The width of the lines connecting the two halves of the plot represents the number of interactions. In the lower half of the plot, specific types of snoRNAs are indicated by distinct colours: SNORA—red, SNORD—blue, and SCARNA—green. In the upper half of the plot, the main categories of interactors are represented by the following colours: transcription factors (TF)—grey, histone modifications—orange, long non-coding RNAs (lncRNA)—light blue, microRNAs (miRNA)—light green, messenger RNAs (mRNA)—black, RNA-binding proteins (RBP)—dark red, and others—pink.

SnoRNAs identified as differentially expressed were analysed in order to identify their major predicted interactors based on the RNAintern database [23]. RNA interactions in this database are detected through a combination of results of experimental methods and computational prediction approaches (e.g., sequence complementarity, structural modelling, motif analysis) and their cross-validation. A dataset encompassing all interactions of each dysregulated snoRNA with a confidence score equal to 0.25 or higher was constructed (Table S2). Among the snoRNAs with altered expression in exosomes following influenza virus infection detected in our study, dysregulated H/ACA box snoRNA (SNORA) were predicted to interact with a total of 304 different interactors (a total of 2034 interactions), C/D box snoRNA with 422 interactors (4884 interactions), and SCARNA with 124 (142 interactions). Based on these data, interactive graphs were constructed separately for SNORA (Figure S1), SNORD (Figure S2), and SCARNA (Figure S3) to visualise the predicted interaction network of each class of snoRNA. It was found that upregulated snoRNAs were predicted to interact mainly with transcription factors (TFs) that were represented by 169 interactors and RNA binding proteins (RBP)—130 interactors. Other categories of interactors included various RNA classes (miRNA, mRNA, lncRNA, and other snoRNA) and proteins involved in histone modification (Figure 2A). In many cases, the single interactor was predicted to be the target of interaction between multiple snoRNAs belonging to different classes. To show dominant interactions for each of the snoRNA classes, a circos plot was constructed (Figure 2B).

The most prominent interactors classified as TF were predicted to interact with more than 50 snoRNAs (Table 2) while multiple were classifed as RBPs with more than 10 snoRNA (Table 3).

## 3. Discussion

The changes in the ncRNA profile observed under pathological conditions include alterations in both cellular and exosomal ncRNAs [25]. The exosomal ncRNA profile is not random: the enrichment of certain ncRNAs indicates that their encapsulation is a regulated biological process that may initiate targeted signalling pathways [26]. In our previous study, we analysed the small RNA composition of exosomes released by IAV-infected and mock-infected MDCK cells using high-throughput sequencing, focusing on differentially expressed protein-coding RNAs (pcRNAs) and pseudogenes. In this study, we provide new insights by expanding our analysis to include differentially expressed snoRNAs. These snoRNAs were systematically classified into three groups—SNORD (C/D box snoRNAs), SNORA (H/ACA box snoRNAs), and SCARNA (snoRNAs with both C/D and H/ACA box features)—based on their structural and functional characteristics using the RNAcentral database. Furthermore, we identified potential interactors for these snoRNAs by applying the RNAinter algorithm, which integrates computational predictions with experimental evidence.

Previous studies suggesting the role of snoRNAs in the pathogenesis of viral diseases primarily focused on the analysis of infected cells rather than secreted exosomes [13].

Murray et al. (2014) in a study involving 12 viruses and different cell lines used gene-trap insertional mutagenesis and gene silencing techniques to show that disrupting the expression of snoRNAs inhibits viral replication. In their research, a total of 83 SNORDs and SNORAs were identified as essential for viral infectivity. A significant number of these snoRNAs were hosted by the *SNHG1*, *SNHG2*, and *TAF1D* genes. The authors described the silencing of these specific snoRNAs as being associated with the inhibition of IAV replication [25]. In our study, we observed that some of these SNORDs were upregulated in exosomes during IAV infection, including SNORD27, SNORD28, and SNORD29, which are hosted by *SNHG1*. In the context of the proposed pro-viral role of this group of snoRNAs, their increased transport via exosomes may reflect the mechanism induced in recipient cells for more efficient virus replication [25].

In vitro studies by Zhuravlev et al. confirmed changes in snoRNA expression in IAV-infected A549 cells [8]. In that study, 66 upregulated and 55 downregulated snoRNAs were identified. Among the snoRNAs that we identified as upregulated in exosomes, there was a group of SNORDs and SNORD families for which Zhuravlev et al. confirmed the downregulation in infected cells (SNORD58ABC, SNORD28, SNORD83A, SNORD73A, SNORD70B, SNORD12, SNORD56, SNORD99, SNORD36AC, SNORD65, SNORD105B, SNORD87, SNORD88B, SNORD21, SNORD46, SNORD59A, SNORD69, SNORD10, and SNORD26). The most downregulated in infected A549 cells was SNORD58A, while in our study, SNORD58 was identified as the most upregulated in exosomes. However, caution should be taken when interpreting this particular discrepancy, as it has been proven that the expression of members of this particular SNORD family is highly dependent on the tissue tested [27]. The studies by Zhuravlev et al. and ours indicate different regulation of some snoRNAs in the cell and exosomes. The negative correlation between cellular and exosomal snoRNA expression may indicate a regulatory mechanism that requires further investigation.

Studies on the pathogenesis of the Chikungunya fever virus and the coronavirus causing COVID-19 have also confirmed differential expression of snoRNAs. During Chikungunya fever virus infection, SNORD3, SNORD44, SNORD76, and SNORD78 (previously called U3, U44, U76, and U78) were upregulated in infected cells [28,29]. Three of these SNORDs are products of the same cluster in the human host gene *GAS5*, whose expression has been linked to apoptosis [30]. The role of *GAS5*-derived snoRNAs in the pathogenesis of various diseases is speculated upon; for instance, SNORD44 is associated with the prognosis of breast cancer or head and neck squamous cell carcinoma [31]. In our experiment, SNORD44 was identified as upregulated in exosomes. Similarly, SNORD44 was identified as the most upregulated in the peripheral blood of patients infected with a severe form of COVID-19 [32]. Parray et al. discovered other snoRNA dysregulations associated with COVID-19 infection; some of these were also identified as upregulated in exosomes in our experiment (SNORA20, SNORD78, SNORD17b, SNORD79) [32]. In our study, one of the two snoRNAs downregulated in exosomes during IAV infection was SNORD3. As described previously, SNORD3 acts as a co-transcriptional molecular chaperone, regulating the excision of rRNAs from the precursor transcript [5]. Its downregulation in exosomes may be important due to its effect on the mRNA “cap-snatching” phenomenon. The viral RNA polymerase complex cleaves 5’-terminal, 10-13 nucleotide-long fragments of host pre-mRNA and uses them to initiate transcription of viral mRNA [14]. This is associated with host protein synthesis “shut-off” and inhibition of cellular gene expression [15]. Studies focused on identifying the sequences of host RNAs cleaved by the influenza virus found that snRNAs and snoRNAs constitute the preferred source of snatched caps [33,34]. An analysis of the snatching rate of the most abundant host RNAs in infected cells (24 h.p.i.) indicated that SNORD3 was among the most frequently involved [33]. In our study, the downregulation of SNORD3 observed in exosomes may be associated with “cap-snatching” in IAV-infected cells. The second downregulated snoRNA, SNORA74, was previously investigated by Qin et al. (2017) [35]. It was shown that silencing of SNORA74B in gallbladder cancer cells led to inhibition of cell proliferation, induced G1 arrest and promoted apoptosis [35]. However, its potential role in viral replication has not yet been established.

When analyzing the interactions of snoRNAs regulated in exosomes (score > 0.25), it can be seen that the dominant groups of their interactors fall into two main categories: transcription factors (TFs) and RNA-binding proteins (RBPs). TFs have been confirmed to control transcription through the coordinated function of DNA and protein-binding domains. TFs have been described as key regulators of cell differentiation and development, as well as being involved in responses to external signals, acting as a link between signalling pathways and gene regulation [33]. In our results, pioneer TFs (POU5F1, SOX2, CEBPB, and MYC) were among the most common interactors for the snoRNAs identified as upregulated in exosomes. Pioneer TFs are a special group of transcription factors that can interact with nucleosomal DNA, “open” closed regions of chromatin, thus enabling the binding of secondary factors, and can initiate regulatory pathways [36]. One of these is POU5F1, which potentially interacts with 76 upregulated snoRNAs identified in our study. The *POU5F1* gene encodes Octamer-binding transcription factor 4 (Oct4), and it has been suggested that the OCT4/miR-125b/BAK1 pathway might be involved in human cervical carcinogenesis [37]. Another main interactor for snoRNA revealed in our analysis was SOX2. The synergistic effect of Sox2-Oct4 in driving the transcription of target genes has already been confirmed, namely, known targets of Sox2-Oct4 are Fgf4, Utf1, and Fbx15, as well as Sox2 itself and Pou5f1. Moreover, it was suggested that this Sox2–Oct4 complex resides at the top of the pluripotent cell’s genetic regulatory network [38]. Herter et al. confirmed that snoRNAs are influenced by MYC and that many are subject to direct transcriptional activation by Myc, both in Drosophila and in vertebrates. Loss of snoRNAs impairs growth during normal development, while their overexpression increases tumour weight in a neuronal tumour model. MYC appears to be a master regulator of snoRNP biogenesis [39]. It was shown that activation of MYC is important for influenza virus replication through the reprogramming of the glutamine catabolism pathway [40].

Some of the pioneer TFs (POU5F1/Oct4, Sox2, MYC), as well as Klf4 (OSKM), are also referred to as “Yamanaka factors”—the core transcriptional factors required to reprogram somatic cells to induce pluripotent stem cells [41]. However, in addition to promoting the formation of pluripotent stem cells, Yamanaka factors have also been implicated in cancer development, also they can affect viral replication and modulation of the immune response. Viruses may alter the levels of these factors to reprogram host cells or to promote their own replication and survival. The interaction of this group of factors with snoRNAs may be important for the course of IAV infection. Hai Feng Wang et al. studied the regulation of Yamanaka factors during H5N1 virus infection in both A549 and HEK293T cells [42]. Their findings provide evidence that the virions and viral proteins of the H5N1 influenza virus regulate the Yamanaka factors through distinct mechanisms. Generally, whole virions had a stronger effect than separated viral proteins. It was confirmed that both NP and PB2 could induce upregulation of OCT4, while KLF4 was only upregulated by NP in A549 cells. They concluded, that expression patterns of Yamanaka factors during H5N1 infection could be a novel regulatory mechanism involved in the pathogenesis of influenza viruses. Influenza virus though snoRNA regulation of Yamanaka factors may exploit pluripotency pathways for their own replication, influencing cell cycle control, apoptosis, and immune evasion.

Viruses use mechanisms to take control of the host gene expression machinery for their own replication. Given the importance of RBPs in processes such as splicing, stability, location, degradation, export, and translation, these proteins are of central interest and may provide control of gene expression resources. Studies indicate that RBP complexes may have a pro- or anti-viral function. Viruses can interact with RBPs to regulate RNA stability and modulate infection [43]. Among the postulated interactors in the RBP category, Polr2a, TNRC6A, IGF2BP3, and FMRP showed the highest number of associations with specific DE snoRNAs. These proteins are all involved in RNA regulation during viral infection. Polr2a, a core component of RNA polymerase II, is responsible for synthesising mRNA, snRNA, and snoRNA. During viral replication, Polr2a can be hijacked to inhibit host mRNA synthesis and facilitate the transcription of viral genes [44]. TNRC6A is part of the RNA-induced silencing complex (RISC) and plays a role in gene regulation by mediating the degradation of host mRNAs, potentially limiting antiviral responses. IGF2BP3 stabilises specific mRNAs, promoting their translation and supporting cell proliferation and survival. It has been suggested that IGF2BP3 inhibits host antiviral innate immunity against RNA viruses by targeting SOCS3 [45]. FMRP interacts with a subset of snoRNAs in the nuclear compartment, and, in the absence of FMRP, rRNA 2’O-methylation is significantly altered, which affects ribosome heterogeneity [46]. FMRP has also been confirmed as a critical host factor utilised by influenza viruses to facilitate the assembly of viral RNPs [47]. Other notable interactors are involved in mRNA methylation (e.g., METTL3, METTL14, YTHDC1, and WTAP), which is particularly important as this modification regulates viral mRNA stability and translation efficiency, aiding in viral replication and immune evasion. Additionally, host splicing factors (e.g., SRSF1 and SRSF7) are co-opted by the virus to facilitate the processing of viral transcripts [48]. In the context of influenza virus infections, HNRNPC and HNRNPK were described as interacting with viral RNAs, potentially influencing their stability and splicing. For example, HNRNPC has been shown to interact with virus RNAs and modulate their processing. Tang et al. confirmed that interaction between HNRNPC exists across different influenza A subtypes and strains. The authors determined that HNRNPC interacts with NP via its C-terminal auxiliary domain and that the HNRNPC is a negative regulator of influenza viral growth. Its interaction with NP is implicated in the promotion of host cell apoptosis during viral infection [49]. Dupont et al. studied RBPs that bind to the viral mRNA encoding the NP of influenza A virus. They showed that TDP-43, encoded by the *TARDBP* gene, binds several influenza mRNAs in addition to NP-mRNA, and that its depletion results in lower levels of viral mRNAs and proteins in infected cells and a reduced yield of infectious viral particles. The authors confirmed that viral polymerase recruits TDP-43 to viral mRNAs through direct interaction with the C-terminal domain of TDP-43 [50]. While there is some evidence for interactions or shared processing mechanisms between identified RBPs and snoRNAs, the precise direct correlations and interactions are still an area of research. These factors may illustrate the complex interaction between the host cellular machinery and viral mechanisms, with each factor likely playing a role in promoting viral replication or modulating the host immune response. Most of the proteins mentioned are better characterised in their roles in mRNA and miRNA regulation; however, they may also play roles in snoRNA biology and can, therefore, influence the course of a viral infection.

When snoRNA upregulation during viral infection occurs in exosomes, this adds another layer of complexity to their potential roles, influencing whether their interactions with RBPs have anti-viral or pro-viral implications. By packaging snoRNAs into exosomes, cells may sequester RBPs or other factors away from viral RNAs, potentially interfering with viral replication. However, the release of snoRNAs in exosomes could help modulate the immune response in recipient cells, possibly suppressing antiviral defences and creating a more favourable environment for the virus. Understanding these dynamics could provide valuable insights into IAV pathogenesis.

## 4. Materials and Methods

### 4.1. Exosome Purification and Small RNA Sequencing

Madin–Darby canine kidney cells were inoculated with IAV A/equi/Kentucky/81 (H3N8) as previously described [22]. Both influenza-infected and mock-infected samples were prepared in duplicate (two biological replicates were used). Briefly, MDCK were cultured in Eagle’s medium containing 10% fetal bovine serum supplemented with Plasmocin (Invivogen, Toulouse, France), and, after a full coverage monolayer was obtained, they were rinsed twice with PBS. Influenza A virus was propagated in 10-day-old specific-pathogen-free (SPF) embryonated chicken eggs, titrated on MDCK cells, and used for inoculation. After 1 h of adsorption, the medium was removed, and the cells were washed twice in PBS and covered with Eagle’s medium without serum. Culture fluids were collected 24 h post-inoculation. In parallel, as a mock-infected control, culture fluids from uninfected MDCK cell cultures were collected. Briefly, a multi-step protocol involving ultracentrifugation and the removal of virions by hemadsorption was used to isolate exosomes. RNA was extracted from exosomes and used for small RNA sequencing and snoRNA identification. The whole procedure was performed as described before [22] and presented in schematic workflow in Figure S4.

### 4.2. High Throughput Sequencing Data Analysis

Differential expression analysis for IAV-infected and mock-infected cell cultures was performed using the DESeq2 R package version 1.34.0 with a linear model based on the negative binomial distribution as described previously [22]. For each transcript, logarithmic fold change (log2FC) was estimated. The significance of log2FC was tested using the Wald test. Multiple testing correction was applied using FDR. Only results with FDR < 0.05 and |log2FC| > 2 were considered significant.

### 4.3. snoRNA Interaction Analysis

Biotypes identified as snoRNA in Ensemble were further divided into SNORD (C/D box snoRNA), SNORA (H/ACA box snoRNA) and SCARNA (C/D and H/ACA box snoRNA) based on RNAcentral: the non-coding RNA sequence database for identification ([51] https://rnacentral.org/ accessed on 22 June 2024). To identify the interactors of over- and under-expressed snoRNAs, the RNA Interactome Database (RNAinter v4.0) was used [23]. For further analysis, only interactors with confidence scores equal to 0.25 or higher and based on experimental evidence were considered. Interactive visualisation of SNORA, SNORD, and SCARNA interaction networks was performed using igraph version 2.0.1 and VisNetwork R version 2.1.2 packages on R Studio Build 446 with R version 4.3.0 [52,53,54]. A circos plot showing the main categories of the interactors for each of the snoRNA types was constructed with circlize version 0.4.15 package [55].

## 5. Conclusions

In this study, we identified significant changes in snoRNA expression profiles in exosomes from influenza-infected cells, with 131 upregulated and 2 downregulated snoRNAs. Notably, several upregulated snoRNAs, such as SNORD27, SNORD28, SNORD29, SNORD58, and SNORD44, have been previously described to be implicated in viral infections, suggesting the role of ncRNAs carried by exosomes during influenza virus replication. Among the most common interactors of upregulated snoRNAs were Transcriptor Factors and RNA-binding proteins. Among interactors within the TF category, we noted that the group belonging to both Yamanaka factors and Pioneer Transcriptions Factors with Oct4/Pou5f1, Sox2, and Myc was the most prominent. They are capable of reprogramming adult somatic cells into pluripotent stem cells, binding to inaccessible chromatin, and initiating the transcription of genes essential for pluripotency. The identification of such interactions for upregulated snoRNAs during viral infection may point to regulatory mechanisms of cell reprogramming occurring during IAV infection. Our findings indicate that snoRNAs may play a pro-viral role during influenza infection. These findings warrant further experimental validation to explore the functional role of the snoRNA in relation to these transcription factors, potentially through knockdown or overexpression studies.

## Figures and Tables

**Table 1:** Number of up- and downregulated snoRNA by category.

snoRNA Category	Upregulated	Downregulated	Total
SNORA	37	1	38
SNORD	92	1	93
SCARNA	2	0	2
All	131	2	133

**Table 2:** Top 20 transcription factors predicted to have the highest number of interactions with snoRNAs dysregulated in exosomes derived from MDCK cells infected with IAV.

	Interactor Name	Interactions			
		SCARNA	SNORA	SNORD	Total
1.	POU5F1	2	20	54	76
2.	SOX2	2	20	53	75
3.	RELA	1	21	49	71
4.	CEBPB	1	18	49	68
5.	AR	2	17	47	66
6.	ESR1	1	16	49	66
7.	MYC	2	19	45	66
8.	SPI1	0	18	48	66
9.	CEBPA	2	16	47	65
10.	PPARG	2	17	46	65
11.	STAT1	1	22	42	65
12.	GATA1	13	48	2	63
13.	ERG	18	41	2	61
14.	KLF4	17	42	0	59
15.	MITF	20	38	1	59
16.	SNAI2	19	39	1	59
17.	CTCF	20	37	1	58
18.	OTX2	10	47	1	58
19.	RUNX1	18	39	1	58
20.	SRF	13	45	0	58

**Table 3:** Top 20 RNA-binding proteins predicted to have the highest number of interactions with snoRNAs dysregulated in exosomes derived from MDCK cells infected with IAV.

	Interactor Name	Interactions			
		SCARNA	SNORA	SNORD	Total
1.	Polr2a	1	15	26	42
2.	TNRC6A	0	1	40	41
3.	IGF2BP3	1	6	25	32
4.	FMR1	2	8	18	28
5.	SRSF1	1	7	17	25
6.	RBFOX2	2	7	14	23
7.	TARDBP	2	4	17	23
8.	DGCR8	1	6	15	22
9.	METTL3	0	6	15	21
10.	METTL14	0	5	12	17
11.	YTHDC1	0	6	10	16
12.	HNRNPC	0	4	10	14
13.	IGF2BP2	0	1	13	14
14.	SRSF7	0	4	10	14
15.	DHX9	1	8	3	12
16.	SRSF3	0	3	9	12
17.	Zfp36	0	5	7	12
18.	HNRNPK	0	5	6	11
19.	LIN28A	0	3	8	11
20.	WTAP	0	8	3	11

## Data Availability

The raw data of the High Throughput Sequencing results have been submitted to NCBI Sequence Read Archive (SRA) under BioProject accession PRJNA904136.

## Supplementary Materials

The supporting information can be downloaded at: https://www.mdpi.com/article/10.3390/ijms26010012/s1.

## Abbreviations

COVID-19	coronavirus disease 19
circRNA	circular RNA
EV	extracellular vesicles
FDR	false discovery rate
IAV	Influenza A Virus
IFN	interferone
log2FC	log2 fold change
lncRNA	long non-coding RNA
mRNA	messenger RNA
MDCK	Madin–Darby canine kidney cells
miRNA	microRNA
ncRNA	non-coding RNA
NP	nucleoprotein
Oct4	octamer-binding transcription factor 4
PB2	polymerase basic protein 2
PBS	phosphate-buffered saline
piRNA	PIWI-interacting RNA
RBP	RNA binding proteins
RISC	RNA-induced silencing complex
rRNA	ribosomal RNA
SCARNA	small Cajal-body specific RNA
sdRNA	sno-derived RNA
snRNA	small nuclear RNA
SNORA	H/ACA box snoRNA
SNORD	C/D box snoRNA
snoRNA	small nucleolar RNA
snoRNP	small nucleolar ribonucleoproteins
SPF	specific-pathogen-free
TERC	telomerase RNA
TF	transcription factors
tRNA	transfer RNA
WNV	West Nile Virus
