# Supplementary material for: Profiling of snoRNAs in Exosomes Secreted from Cells Infected with Influenza A Virus
Source: Int J Mol Sci. 2024 Dec 24;26(1):12. doi: 10.3390/ijms26010012 (PMC11720657; doi:10.3390/ijms26010012)
Supplement: Supplementary file 1 [file ijms-26-00012-s001.zip › Supplementary captions.pdf]

Figure S1. Interaction network of 38 SNORAs dysregulated in exosomes from IAV infected cells. “Select by id” - allows to selected particular interactors (including snoRNA) while “Select by group” – allows to select groups of interactors belonging to selected category. Visualization was constructed using using igraph and VisNetwork R packages with R version 4.3.0.

Figure S2. Interaction network of 93 SNORDs dysregulated in exosomes from IAV infected cells. “Select by id” - allows to selected particular interactors (including snoRNA) while “Select by group” – allows to select groups of interactors belonging to selected category. Visualization was constructed using using igraph and VisNetwork R packages with R version 4.3.0.

Figure S3. Interaction network of 2 SCARNAs dysregulated in exosomes from IAV infected cells. “Select by id” - allows to selected particular interactors (including snoRNA) while “Select by group” – allows to select groups of interactors belonging to selected category. Visualization was constructed using using igraph and VisNetwork R packages with R version 4.3.0.

Figure S4. Chart showing main experimental steps.

Table S1. Rank of differently expressed snoRNAs in exosomes from IAV infected MDCK cells.

Table S2. SnoRNA interactions.
