# Supplementary material for: Profiling of snoRNAs in Exosomes Secreted from Cells Infected with Influenza A Virus
Source: Int J Mol Sci. 2024 Dec 24;26(1):12. doi: 10.3390/ijms26010012 (PMC11720657; doi:10.3390/ijms26010012)
Supplement: Supplementary file 1 [file ijms-26-00012-s001.zip › Table S1.pdf]

Table S1. Rank of differently expressed snoRNAs in exosomes from IAV infected MDCK cells

Rank	Ensambl ID (v97)	Rfam	snoRNA	padj	log2 FoldChange	snoRNA class
1	ENSCAFG00000021934	RF00151	SNORD58	2,66E-08	9,61	C/D box
2	ENSCAFG00000021357	RF00279	SNORD45	1,16E-05	8,25	C/D box
3	ENSCAFG00000021294	RF00341	Z39	2,89E-05	8,04	C/D box
4	ENSCAFG00000026308	RF00085	SNORD28	2,95E-05	7,92	C/D box
5	ENSCAFG00000028034	RF01277	SNORD54	5,89E-05	7,74	C/D box
6	ENSCAFG00000030924	RF00108	SNORD116	0,000164715	7,50	C/D box
7	ENSCAFG00000025947	RF00270	SNORD61	0,000305949	7,36	C/D box
8	ENSCAFG00000020981	RF00137	SNORD83	0,000269623	7,33	C/D box
9	ENSCAFG00000025815	RF00071	SNORD73	0,000422024	7,20	C/D box
10	ENSCAFG00000026205	RF00575	SNORD70	0,000684662	7,07	C/D box
11	ENSCAFG00000026458	RF00154	SNORD63	0,00080869	7,02	C/D box
12	ENSCAFG00000032651	RF00188	SNORD103	0,00168841	6,84	C/D Box
13	ENSCAFG00000025886	RF00151	SNORD58	0,001863902	6,75	C/D box
14	ENSCAFG00000027004	RF00569	SNORD19	0,000358455	6,73	C/D box
15	ENSCAFG00000025841	RF00151	SNORD58	1,01E-06	6,70	C/D box
16	ENSCAFG00000031022	RF00188	SNORD103	8,18E-37	6,56	C/D box
17	ENSCAFG00000025790	RF00581	SNORD12/SNORD106	1,56E-06	6,46	C/D box
18	ENSCAFG00000025916	RF00086	SNORD27	4,84E-21	6,22	C/D box
19	ENSCAFG00000026287	RF00581	SNORD12/SNORD106	9,04E-06	6,15	C/D box
20	ENSCAFG00000022413	RF00091	SNORA62/SNORA6	8,94E-06	6,15	H/ACA box
21	ENSCAFG00000026135	RF00092	SNORA63	0,014714547	6,09	H/ACA box
22	ENSCAFG00000025859	RF00275	SNORD56	0,038788768	6,06	C/D box
23	ENSCAFG00000026346	RF00569	SNORD19	0,016560864	6,02	C/D box
24	ENSCAFG00000028271	RF01191	SNORD121A	0,020404883	5,94	C/D box
25	ENSCAFG00000028124	RF00581	SNORD12	5,07E-17	5,94	C/D box
26	ENSCAFG00000028095	RF01200	SNORD125	0,022241844	5,93	C/D box
27	ENSCAFG00000025882	RF00591	SNORD77	1,82E-14	5,72	C/D box
28	ENSCAFG00000027378	RF00576	SNORD71	0,038683605	5,68	H/ACA box

29	ENSCAFG00000021073	RF00070	SNORD29	1,76E-09	5,67	C/D box
30	ENSCAFG00000026013	RF00091	SNORA62/SNORA6	9,97E-07	5,45	H/ACA box
31	ENSCAFG00000020926	RF00413	SNORA19	0,000537601	5,44	H/ACA box
32	ENSCAFG00000027802	RF01295	SCARNA7	6,37E-18	5,40	C/D box and H/ACA box
33	ENSCAFG00000025984	RF00153	SNORD62	1,44E-05	5,37	C/D box
34	ENSCAFG00000027472	RF00136	SNORD81	5,33E-08	5,31	C/D box
35	ENSCAFG00000026679	RF00608	SNORD99	2,81E-05	5,21	C/D box
36	ENSCAFG00000021344	RF00136	SNORD81	1,25E-15	5,18	C/D box
37	ENSCAFG00000026464	RF00072	SNORA75	8,59E-16	5,16	H/ACA box
38	ENSCAFG00000025857	RF00186	SNORD101	3,87E-05	5,16	C/D box
39	ENSCAFG00000026367	RF00287	SNORD44	5,02E-18	5,07	C/D box
40	ENSCAFG00000025748	RF00187	SNORD102	7,86E-14	5,02	C/D box
41	ENSCAFG00000026335	RF00147	SNORD34	1,14E-17	5,00	C/D box
42	ENSCAFG00000026113	RF00049	SNORD36	1,99E-07	4,97	C/D box
43	ENSCAFG00000028262	RF01291	SNORD97	1,69E-08	4,93	C/D box
44	ENSCAFG00000026284	RF00137	SNORD83	1,71E-07	4,90	C/D box
45	ENSCAFG00000022659	RF00281	SNORD47	4,74E-11	4,88	C/D box
46	ENSCAFG00000026011	RF00571	SNORD65	1,40E-13	4,82	C/D box
47	ENSCAFG00000026801	RF00603	SNORD23	0,003554363	4,81	C/D box
48	ENSCAFG00000028078	RF01161	SNORD5	8,64E-08	4,69	C/D box
49	ENSCAFG00000026374	RF00563	SNORA53	3,41E-14	4,66	H/ACA box
50	ENSCAFG00000021628	RF00265	SNORA69	5,31E-13	4,58	H/ACA box
51	ENSCAFG00000025775	RF00402	SNORA25	7,29E-09	4,56	H/ACA box
52	ENSCAFG00000027974	RF01183	SNORD19B	0,012643155	4,45	C/D box
53	ENSCAFG00000026199	RF00577	SNORD72	2,03E-10	4,44	C/D box
54	ENSCAFG00000021569	RF00279	SNORD45	0,002392702	4,21	C/D box
55	ENSCAFG00000028047	RF00570	SNORD64	2,30E-11	4,21	C/D box
56	ENSCAFG00000022368	RF00091	SNORA62/SNORA6	1,01E-12	4,21	H/ACA box
57	ENSCAFG00000025856	RF00560	SNORA17	4,09E-11	4,18	H/ACA box
58	ENSCAFG00000027576	RF01182	SNORD11	0,001166172	4,15	C/D box

59	ENSCAFG00000028433	RF01210	U13	1,50E-05	4,09	C/D box
60	ENSCAFG00000026209	RF00069	SNORD24	1,97E-10	4,06	C/D box
61	ENSCAFG00000025879	RF00584	SNORD105	5,37E-07	3,99	C/D box
62	ENSCAFG00000022187	RF00439	SNORD87	0,000189512	3,93	C/D box
63	ENSCAFG00000026247	RF00592	SNORD78	1,61E-08	3,91	C/D box
64	ENSCAFG00000025938	RF00416	SNORA43	1,59E-12	3,88	H/ACA box
65	ENSCAFG00000028099	RF00401	SNORA20	1,04E-07	3,88	H/ACA box
66	ENSCAFG00000022406	RF00016	SNORD14	8,10E-09	3,85	C/D box
67	ENSCAFG00000020852	RF00438	SNORA33	7,58E-10	3,85	H/ACA box
68	ENSCAFG00000026788	RF00604	SNORD88	1,38E-06	3,84	C/D box
69	ENSCAFG00000020849	RF00398	SNORA15	3,79E-05	3,80	H/ACA box
70	ENSCAFG00000026603	RF00612	SNORD75	3,11E-07	3,77	C/D box
71	ENSCAFG00000025861	RF00211	SNORD35	0,000422765	3,75	C/D box
72	ENSCAFG00000029001	RF00108	SNORD116	0,003248186	3,75	C/D box
73	ENSCAFG00000026275	RF00016	SNORD14	1,38E-09	3,72	C/D box
74	ENSCAFG00000025830	RF00189	SNORD95	5,54E-07	3,69	C/D box
75	ENSCAFG00000025773	RF00429	SNORA29	4,36E-05	3,59	H/ACA box
76	ENSCAFG00000022076	RF00067	SNORD15-201	7,13E-08	3,59	C/D box
77	ENSCAFG00000029523	RF00157	SNORD39	1,04E-07	3,58	C/D box
78	ENSCAFG00000020742	RF00068	SNORD21	1,59E-08	3,57	C/D box
79	ENSCAFG00000026445	RF00218	SNORD46	8,10E-05	3,51	C/D box
80	ENSCAFG00000021068	RF00406	SNORA42/SNORA80	2,58E-10	3,44	H/ACA box
81	ENSCAFG00000025786	RF00016	SNORD14	5,92E-09	3,43	C/D box
82	ENSCAFG00000025798	RF00289	SNORD104	2,97E-06	3,38	C/D box
83	ENSCAFG00000021715	RF00273	SNORD59	0,000369713	3,37	C/D box
84	ENSCAFG00000021275	RF00213	R38	0,000197358	3,31	C/D box
85	ENSCAFG00000026039	RF00418	SNORA58	5,00E-07	3,31	H/ACA box
86	ENSCAFG00000022282	RF00278	SNORD50	8,26E-05	3,26	C/D box
87	ENSCAFG00000021640	RF00138	SNORD16	0,005022746	3,22	C/D box
88	ENSCAFG00000025973	RF00591	SNORD77	1,83E-05	3,20	C/D box
89	ENSCAFG00000025787	RF00432	SNORA51	1,98E-08	3,18	H/ACA box

90	ENSCAFG00000027602	RF01192	SNORD11B	0,032553718	3,16	C/D box
91	ENSCAFG00000025846	RF00049	SNORD36-201	1,93E-05	3,15	C/D box
92	ENSCAFG00000027518	RF00478	SCARNA6	1,85E-05	3,14	C/D box and H/ACA box
93	ENSCAFG00000025925	RF00567	SNORD17	6,05E-07	3,13	C/D box
94	ENSCAFG00000028434	RF01210	U13	1,36E-06	3,12	C/D box
95	ENSCAFG00000021975	RF00091	SNORA62/SNORA6	0,000111626	3,05	H/ACA box
96	ENSCAFG00000026472	RF00278	SNORD50	1,12E-05	3,05	C/D box
97	ENSCAFG00000026040	RF00580	SNORD91	0,004863248	3,04	C/D box
98	ENSCAFG00000028033	RF01233	U109	3,90E-05	3,03	H/ACA box
99	ENSCAFG00000027561	RF01164	SNORD107	0,010898098	3,01	C/D box
100	ENSCAFG00000020948	RF00069	SNORD24	0,001531393	3,00	C/D box
101	ENSCAFG00000026246	RF00212	SNORD38	2,57E-05	2,94	C/D box
102	ENSCAFG00000021886	RF00324	MBII-202RNA	0,000127791	2,90	C/D box
103	ENSCAFG00000026348	RF00535	snoMe28S-Am982	0,000186957	2,85	C/D box
104	ENSCAFG00000025814	RF00593	SNORD83B	4,19E-07	2,83	C/D box
105	ENSCAFG00000020859	RF00276	SNORD52	0,000795067	2,81	C/D box
106	ENSCAFG00000026385	RF00152	SNORD79	3,07E-05	2,79	C/D box
107	ENSCAFG00000022704	RF00399	SNORA24	2,49E-06	2,77	H/ACA box
108	ENSCAFG00000025991	RF00420	SNORA61	1,45E-06	2,75	H/ACA box
109	ENSCAFG00000026279	RF00574	SNORD69	0,00682224	2,74	C/D box
110	ENSCAFG00000021944	RF00211	SNORD35	0,020478433	2,69	C/D box
111	ENSCAFG00000021151	RF00092	SNORA63	3,28E-07	2,64	H/ACA box
112	ENSCAFG00000027886	RF01290	SNORD10	9,94E-05	2,64	C/D box
113	ENSCAFG00000026225	RF00087	SNORD26	0,000842189	2,48	C/D box
114	ENSCAFG00000026616	RF00614	SNORA11	0,021333523	2,47	H/ACA box
115	ENSCAFG00000025907	RF00416	SNORA43	0,000922956	2,46	H/ACA box
116	ENSCAFG00000026322	RF00263	SNORA68	2,28E-05	2,45	H/ACA box
117	ENSCAFG00000025838	RF00056	SNORA71	3,15E-05	2,41	H/ACA box
118	ENSCAFG00000026033	RF00334	SNORA3/SNORA45	0,000582337	2,34	H/ACA box
119	ENSCAFG00000022178	RF00133	Z195/SNORD33/SNORD32	0,000394661	2,33	C/D box

120	ENSCAFG00000022498	RF00421	SNORA32	0,0002542	2,30	H/ACA box
121	ENSCAFG00000027038	RF00413	SNORA19	0,000327025	2,29	H/ACA box
122	ENSCAFG00000026131	RF00562	SNORA49	0,00154896	2,22	H/ACA box
123	ENSCAFG00000026736	RF00611	SNORD111	0,034464663	2,21	C/D box
124	ENSCAFG00000026350	RF00071	SNORD73	0,000947952	2,19	C/D box
125	ENSCAFG00000026675	RF00599	SNORA77	0,008527044	2,18	H/ACA box
126	ENSCAFG00000026245	RF00099	SNORD22	1,58E-05	2,15	C/D box
127	ENSCAFG00000022333	RF00302	SNORA65	0,001956452	2,11	H/ACA box
128	ENSCAFG00000025831	RF00158	SNORD82	0,00839826	2,06	C/D box
129	ENSCAFG00000026193	RF00560	SNORA17	0,008855213	2,06	H/ACA box
130	ENSCAFG00000026676	RF00614	SNORA11	0,032993326	2,06	H/ACA box
131	ENSCAFG00000027581	RF01241	SNORA81	0,000110416	2,00	H/ACA box
132	ENSCAFG00000025850	RF00090	SNORA74	0,000134376	-2,67	H/ACA box
133	ENSCAFG00000021718	RF00012	U3	1,29E-08	-2,98	C/D box
