# Supplementary figures and images for: Profiling of snoRNAs in Exosomes Secreted from Cells Infected with Influenza A Virus
Source: Int J Mol Sci. 2024 Dec 24;26(1):12. doi: 10.3390/ijms26010012 (PMC11720657; doi:10.3390/ijms26010012)

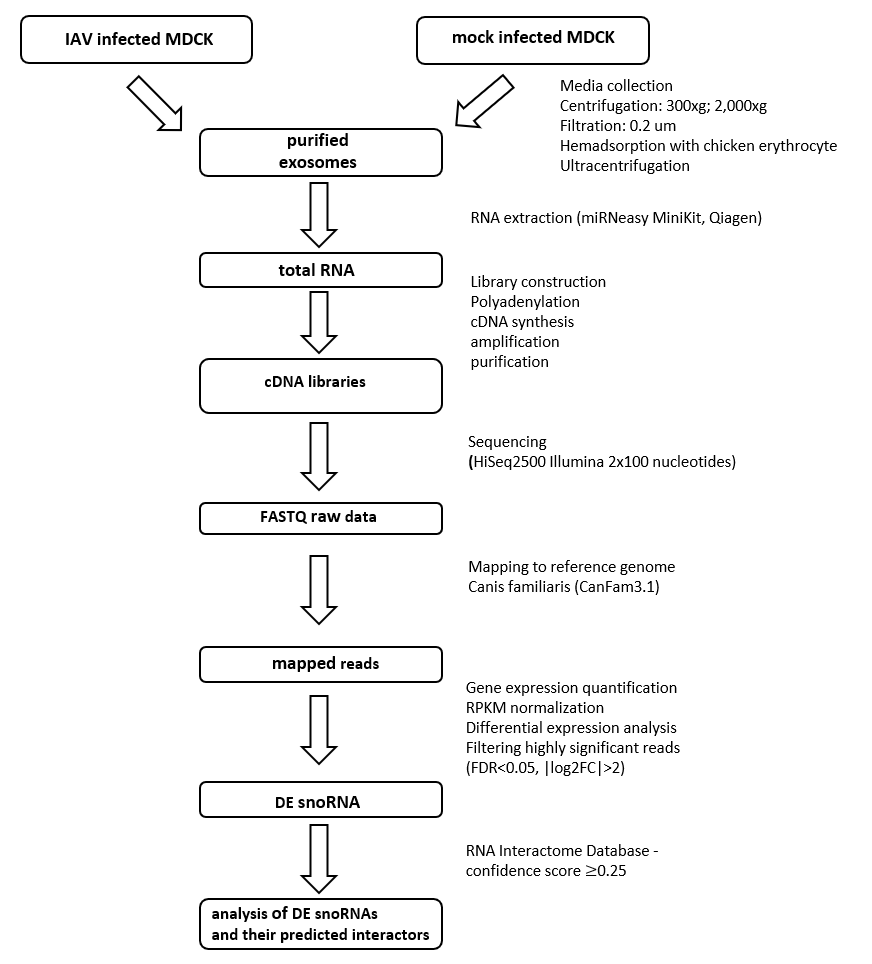

Supplement: Supplementary file 1 [file ijms-26-00012-s001.zip › Figure S4.tif]
